# Interactive Influence of Item Competitive Strength and Inhibition Ability on Retrieval‐Induced Forgetting

**DOI:** 10.1002/pchj.70007

**Published:** 2025-03-10

**Authors:** Yue Chu, Hui Xu, Weihai Tang, Xiping Liu

**Affiliations:** ^1^ Faculty of Psychology Tianjin Normal University Tianjin China; ^2^ School of Sociology University of Sanya Sanya Hainan China

**Keywords:** inhibition ability, item competitive strength, retrieval‐induced forgetting

## Abstract

Retrieval‐induced forgetting (RIF) occurs when selective retrieval of certain information leads to the forgetting of other related information. Previous studies have shown that individuals with varying inhibition abilities can exhibit similar RIF magnitudes, a finding not entirely consistent with existing theories. This study aimed to investigate the interaction between item competitive strength and inhibition ability in modulating RIF. Items were categorized into high‐, medium‐, and low‐competitive strength groups based on taxonomic frequency ratings. Participants' inhibition abilities were assessed using the Stroop task, and RIF was examined across these groups. The results revealed that at high‐item competitive strength, only high‐inhibition participants showed RIF. At medium item competitive strength, both groups demonstrated RIF, with no difference in magnitude. At low‐item competitive strength, neither group exhibited RIF. These findings suggest that both item competitive strength and inhibition ability modulate RIF, supporting the inhibition theory of RIF.

## 1 Introduction

The retrieval of information has a constraining effect on subsequent memory retention. One of the typical effects is called retrieval‐induced forgetting (RIF) (Anderson et al. 1994; Buchli 2023). When individuals selectively retrieve parts of information from memory, it can lead to forgetting other relevant information.

RIF is typically examined using the retrieval practice paradigm. The paradigm typically consists of four phases: Study phase, retrieval practice phase, distractor phase, and test phase. In the study phase, participants study a series of category‐exemplar pairs, for example, “Fruit—apple”, “Fruit—strawberry”, “Animal—tiger”. In the retrieval practice phase, participants practice retrieving some of the exemplars from certain categories through the presentation of category‐plus‐stem (e.g., Fruit—ap___). After that, a distractor phase follows. During the final test phase, participants are required to recall all category‐exemplar pairs from the study phase. This paradigm creates three types of items: Exemplars receiving retrieval practice (e.g., Rp+ items; apple), exemplars that belong to the same categories as practiced examples but are not actually practiced (e.g., Rp− items; strawberry), and unpracticed exemplars from unpracticed categories (e.g., Nrp items; tiger). The recall of Nrp items is better than that of Rp− items, a phenomenon known as RIF (Anderson et al. 1994; Reeck and LaBar 2024; Rupprecht and Bäuml 2016).

**FIGURE 1:**
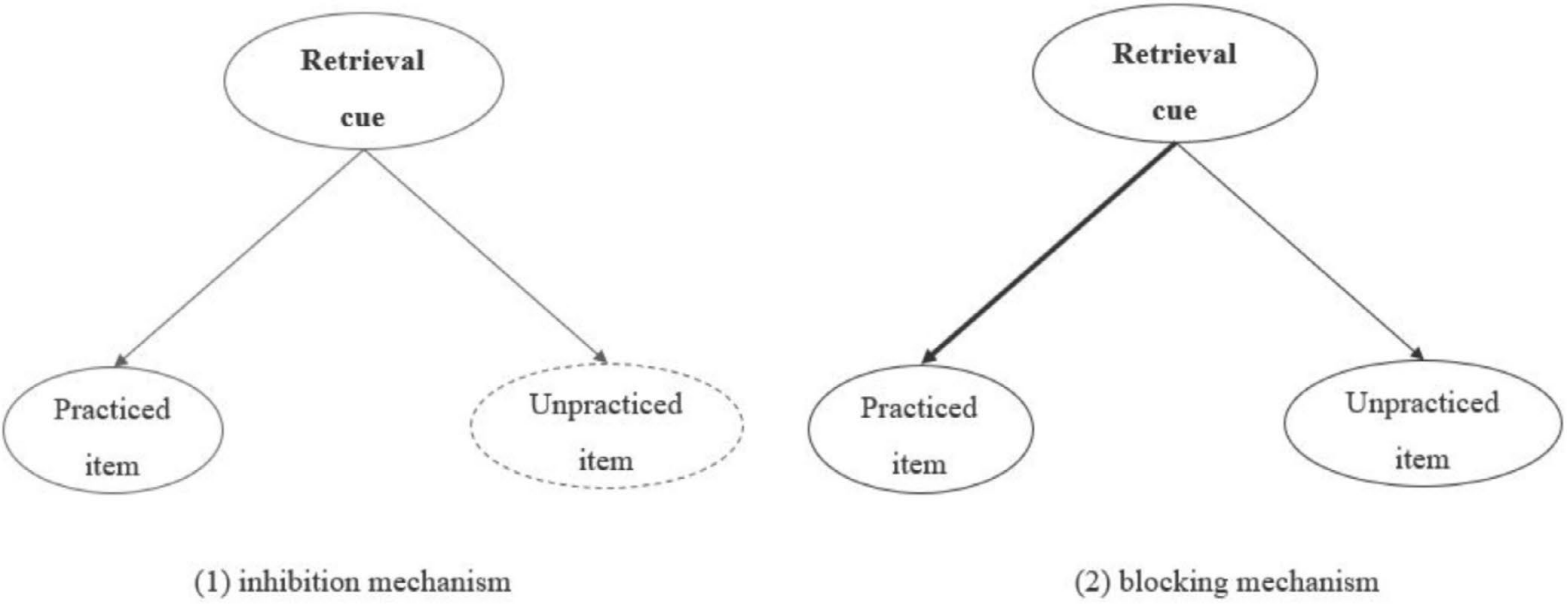
Illustration of the inhibition and blocking mechanisms.

Numerous studies have explored the mechanisms of RIF (Anderson et al. 1994; Román et al. 2009; Rupprecht and Bäuml 2016; Verde 2013; for a review, see Murayama et al. 2014). There are two different accounts: The inhibition theory and the blocking theory (Bäuml and Kliegl 2017; Marsh and Anderson 2024) (Figure 1). The inhibition theory argues that the RIF is caused by an inhibition mechanism, and the target items are subject to competition from nontarget items during the retrieval practice phase. In order to minimize this competition and enhance the retrieval of target items, nontarget items are inhibited. This weakens memory representations of nontarget items and causes subsequent memory impairment for these items (Anderson 2003; Kovacs and Harris 2022; Murayama et al. 2014). According to the blocking theory, which is based on strength‐dependent competition models, retrieval practice strengthens the association between target items and category cues. This enhanced association leads to the blocking of the recall of nontarget items during the test phase, ultimately decreasing the recall of nontarget items (Raaijmakers and Jakab 2013; Zhang et al. 2023).

The inhibition and blocking theories differ primarily in two aspects: The phases during which they operate and their impacts on nontarget items. During the retrieval practice phase, the inhibition mechanism inhibits nontarget items while weakening their memory representations, an effect that persists across diverse memory tests. Conversely, the blocking mechanism operates during the testing phase, where the enhanced association between target items and cues blocks the retrieval of nontarget items. Blocking does not influence the association between the nontarget items and their retrieval cue, nor does it impact the memory representation of the nontarget items. Based on these two distinctions, the contributions of inhibition and blocking mechanisms vary depending on the type of test employed. If the tests eliminate the role of “association strength,” the blocking mechanism may have difficulty functioning. Cued recall and recognition tests are frequently utilized within the retrieval practice paradigm, with RIF commonly attributed to both inhibition and blocking processes in cued recall. Nonetheless, in item recognition tasks, interference from other items is typically minimal, rendering the blocking mechanism less operative in such tests (Rupprecht and Bäuml 2016; Shiffrin and Steyvers 1997). Thus, employing recognition tests allows for the examination of these two mechanisms within the retrieval practice paradigm. Despite debates surrounding the inhibition and blocking mechanisms, the two‐factor theory has been posited to account for RIF, suggesting the inhibition and blocking work together (Rupprecht and Bäuml 2016; Schilling et al. 2014). The two‐factor theory offers a comprehensive framework for explaining a wide range of RIF phenomena. However, as an overarching concept, it encompasses several critical issues that warrant further elucidation. For instance, it is essential to determine the conditions under which the two mechanisms simultaneously operate. More empirical studies are needed in the future to address these critical issues.

A substantial body of research in behavioral and neuroimaging studies has provided evidence for the inhibition mechanism underlying RIF (Agarwal et al. 2017; Grundgeiger 2014; Kuhl et al. 2007; Rupprecht and Bäuml 2016; Kozlovskiy et al. 2023; Storm and Angello 2010; Wimber et al. 2011). Cognitive inhibition reflects the ability to actively inhibit and limit the processing of stimuli unrelated to the current task (Anderson and Levy 2009). It is precisely because nontarget items are inhibited during the retrieval practice that memory impairment is manifested. This has led researchers to pay great attention to the relationship between individual inhibition ability and RIF. These studies typically involve categorizing participants based on their inhibition abilities into high and low groups, comparing the RIF performance between groups with different inhibition abilities (Liu et al. 2019; Zhang et al. 2023). Additionally, some research examines whether there is a positive correlation between the level of inhibition ability and the magnitude of RIF (Noreen and MacLeod 2015; Schilling et al. 2014), whereas others have focused on whether individuals with inhibition deficits exhibit RIF comparable to those observed in individuals with typical inhibition abilities (Soriano et al. 2009; Storm and White 2010). From the perspective of inhibition theory, individuals with stronger inhibition abilities are expected to exhibit a more pronounced RIF effect. Empirical evidence supports this prediction, indicating that those with heightened inhibition control are more likely to experience a greater RIF compared with individuals with weaker inhibition (Aslan and Bäuml 2011, 2012; Román et al. 2009). Moreover, research has identified a positive correlation between inhibition ability and RIF effect, suggesting that enhanced inhibitory control is associated with a more significant RIF (Schilling et al. 2014; Storm and Bui 2016). However, this prediction is not universally supported. A subset of studies has observed that individuals with varying levels of inhibition ability display equivalent RIF magnitudes (Aslan and Bäuml 2010; Ford et al. 2004; Soriano et al. 2009; Storm and White 2010). These studies can be classified into two categories based on the tests employed. One category includes cued recall, which permits the concurrent operation of inhibition and blocking mechanisms. The other category involves recognition tests, where blocking mechanism is less effective, and RIF is primarily a result of inhibition processes.

In light of the findings from cued recall studies, researchers have posited the view of *the correlated costs and benefits problem* to elucidate these outcomes (Anderson and Levy 2007). It posits that individuals with stronger inhibition abilities show greater resistance to interference. Within the retrieval practice paradigm, those with high‐inhibition abilities are capable of inhibiting Rp− items during retrieval and resisting blocking during testing. Conversely, individuals with low‐inhibition abilities fail to inhibit Rp− items during retrieval and are unable to prevent blocking in testing. For individuals with high‐inhibition abilities, RIF is attributed to the inhibition mechanism, whereas in those with low abilities, it is ascribed to the blocking mechanism. This suggests that although individuals with varying inhibition abilities may exhibit similar RIF magnitudes, the underlying mechanisms may differ. Studies have shown that individuals with weaker inhibition control, such as those with schizophrenia or ADHD, exhibit comparable RIF magnitudes to controls in category‐cued recall, with no RIF in recognition tests or category‐plus‐stem recall (Soriano et al. 2009; Storm and White 2010). In brief, within these studies, individuals with high‐inhibition ability show RIF through the inhibition mechanism, whereas those with low‐inhibition ability cannot rely on the inhibition mechanism to show RIF but instead rely on the blocking mechanism, resulting in equivalent RIF levels between high‐ and low‐inhibition ability individuals. This is not contradictory to the inhibition mechanism.

However, when recognition tests are employed, it is still possible to observe that individuals with varying levels of inhibition ability exhibit equivalent levels of RIF. Studies have reported that individuals with schizophrenia exhibit RIF comparable to the control group in recognition tasks (AhnAllen et al. 2007). Additionally, Ford et al. (2004) found that 7‐year‐old children and young adults displayed equivalent RIF magnitudes in recognition tests. These research findings do not align with the predictions of the inhibition mechanism, and they are also inexplicable from the view of *the correlated costs and benefits problem*. Explaining the results observed in recognition tests is a question that warrants exploration.

The competition dependence of RIF is one of the properties of inhibition theory, indicating that only nontarget items that compete with the retrieval of the target items are inhibited, rather than all nontarget items (Anderson 2003; Bäuml and Kliegl 2017). RIF is influenced by the amount of competition caused by nontarget items, as supported by several studies. In these studies, researchers classified exemplars into high‐ and low‐taxonomic frequency exemplars, finding that RIF was only observed in the high‐taxonomic frequency exemplars (Anderson et al. 1994; Bai and Liu 2014; Migueles and García‐Bajos 2014). This is because high‐taxonomic frequency exemplars are more likely to be activated based on category cues, competing for the retrieval of target items, and as such, they are more susceptible to inhibition. We defined this amount of competition from nontarget items as item competitive strength. Item competitive strength is recognized as a key determinant of RIF. Prior studies have often overlooked this factor when analyzing the link between inhibition ability and RIF. Does RIF differ between individuals with high‐ versus low‐inhibition abilities under varying item competitive strength?

The study aimed to investigate how item competitive strength, in conjunction with inhibition ability, influences RIF. The interaction between the two factors may explain inconsistencies in RIF across individuals with different inhibition abilities, offering a new perspective on previous findings. Following Anderson et al. (1994), we manipulated item taxonomic frequency to manage competition from nontarget to target items and employed a recognition test within the retrieval practice paradigm. The Stroop task, a well‐established measure of inhibition control, has been extensively utilized in cognitive research (Comalli Jr. et al. 1962; Okayasu et al. 2023; Stroop 1935). It has also been employed in RIF studies to assess inhibition abilities (Ciaramella 2018; Noreen and MacLeod 2015; Wimber et al. 2009). Our study similarly applied the Stroop task to assess participants' inhibition abilities. We hypothesized that at low‐item competitive strength, where competition from nontarget items was minimal, both high‐ and low‐inhibition individuals would not demonstrate RIF. In contrast, at medium item competitive strength, individuals with high‐ and low‐inhibition abilities were expected to demonstrate similar magnitudes of RIF as they effectively handled competition from nontarget items. Finally, at high‐item competitive strength, individuals with high‐inhibition ability were expected to demonstrate a greater magnitude of RIF than those with low‐inhibition ability. Consistent results with our hypothesis would explain the equal RIF observed across individuals with different inhibition control in recognition tests.

## 2 Methods

### 2.1 Participants

We advertised to recruit non psychology students for a study with payment upon completion. Initially, 70 participants enrolled, but five were excluded: Three for longer response times in the congruent condition of the Stroop task and two for extreme values of the interference effect (e.g., three standard deviations from the mean). The final sample included 65 participants. We ranked participants by their Stroop interference effect and categorized the top 27% (criterion of 27%, referring to Kelley 1939; Ramu et al. 2023) as the high‐inhibition group (1 male, *M*_age_ = 19.67 ± 1.14) and the bottom 27% as the low‐inhibition group (2 males, *M*_age_ = 20.06 ± 1.63), each comprising 18 individuals. Based on the hypothesis that a stable RIF would be evident at medium item competitive strength, we utilized G*Power 3.1 to calculate the necessary sample size. With an effect size (*f*) of 0.25, a power (1−*β*) of 80%, and an alpha level of 5%, it was determined that 14 participants per group were required to achieve adequate statistical power for the analysis. The sample size of this study met the requirement.

### 2.2 Design

In the study, item competitive strength (high, medium, low) and item type (Rp+, Rp−, Nrp) were manipulated as within‐subjects variables, whereas inhibition ability was a between‐subjects variable. Recognition performance was measured by the correct hit rates for exemplars. The study aimed to examine the RIF performance of individuals with high‐ versus low‐inhibition abilities across varying levels of item competitive strength. Consistent with this aim, the results section presented a separate analysis of participants' memory performance at each item competitive strength.

### 2.3 Materials

#### 2.3.1 RIF Materials

##### 2.3.1.1 Study Materials

Following Battig and Montague (1969), eight categories were selected, with six serving as retrieval practice (Fruit, Sport, Topography, Disease, Occupation, Flower) and two as baselines (Crime, Body), each comprising 36 exemplars. The following principles guided the selection of materials: Choosing relatively unrelated categories, selecting semantically clear Chinese two‐character words, and ensuring the first word of the exemplars under each category was unique. Forty‐eight psychology students evaluated the taxonomic frequency of each exemplar on a 7‐point scale, with “1” as the least frequent and “7” as the most, using the prompt: “When presented with a category, how quickly can you come up with the exemplar?” (e.g., “Piano” for “Musical Instrument”). The exemplars in each category were ranked from high to low based on their average ratings and divided into three groups, with 12 exemplars in each group. Group 1 included positions 1–12, Group 2 positions 13–24, and Group 3 positions 25–36, with significant rating differences observed among groups within each category (The study materials can be found in Supporting Information S3).

Prior research suggests high‐taxonomic frequency items are more competitive. We categorized items into high‐, medium‐, and low‐competitive strength based on frequency ratings, with significant differences observed: Occupation and Sport (high), Flower and Fruit (medium), and Disease and Topography (low). Four exemplars from each subgroup of the baseline categories were selected, and Nrp items were also classified by strength. The ratings across strength are shown in Table 1. The study materials consisted of eight categories (96 exemplars total), randomly divided into six blocks, each containing one exemplar from each category (e.g., “Occupation—doctor”), with two pairs of unrelated fillers before and after the study phase. (The results of the rating comparisons can be found in Supporting Information S1 and S2).

**TABLE 1:** Ratings of different types of item under each strength (mean ± SD).

	High strength	Medium strength	Low strength
Rp+	6.43 ± 0.26	5.46 ± 0.35	4.01 ± 0.33
Rp−	6.05 ± 0.16	4.80 ± 0.45	3.28 ± 0.38
Nrp	6.29 ± 0.17	5.56 ± 0.43	4.26 ± 1.13

##### 2.3.1.2 Retrieval Materials

Practice materials consisted of category‐plus‐stem pairs (e.g., “Occupation—do( )”). Half of the exemplars from six retrieval categories (36 total) were practiced, with each exemplar repeated three times across three blocks, ensuring no consecutive exemplars from the same category.

##### 2.3.1.3 Test Materials

The recognition test included 192 exemplars, half from the study phase and half lures, organized into 12 blocks, each containing one old and one new exemplar per category.

#### 2.3.2 Stroop Task

The Stroop color‐word task included congruent (e.g., “green” in green ink) and incongruent (e.g., “red” in green ink) conditions using the words red, yellow, blue, and green in matching inks (Vasta et al. 2024). The task had a practice phase (8 trials) and an experimental phase (96 trials), with equal congruent and incongruent trials. Each trial began with a 250 ms fixation, followed by the color‐word stimulus. Participants indicated the ink color using keys (D/F/J/K for red/green/yellow/blue). The interference effect was calculated as the response time difference between congruent and incongruent conditions, with larger effects indicating weaker inhibition. Reaction times were recorded for all trials.

#### 2.3.3 Equipment

A laptop computer, with the experiment administered using E‐Prime 2.0 (computer monitor size: 13.5 in.; resolution: 1280 × 768 pixels).

### 2.4 Procedures

**FIGURE 2:**
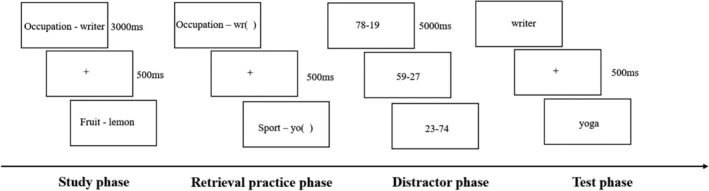
Procedures for RIF paradigm.

Participants first completed the Stroop task, followed by the RIF task, which consisted of four stages. In the study phase, exemplars were randomly presented for 3 s per block, with instructions to memorize the pairs for a subsequent memory test. During the retrieval practice phase, participants completed category‐exemplar stems verbally, based on their recall, with performance recorded; they proceeded by button press or after a 5 s timeout. The distractor phase involved solving 36 subtraction problems over 3 min, with each displayed for 5 s and answers documented. The test phase was a recognition test distinguishing “old” from “new” exemplars, with “*Z*” for old and “*M*” for new; items advanced postresponse or after a 5 s interval. Participants had practice trials prior to the formal test (Figure 2).

## 3 Results

### 3.1 Inhibition Ability

An independent samples *t*‐test revealed a significant difference in the interference effect between the high‐ and low‐inhibition groups, *t* (34) = −13.01, *p <* 0.001, Cohen's *d* = −4.34, suggesting that the high inhibition group showed stronger inhibition control (Table 2).

**TABLE 2:** The interference effect in the high‐ and low‐inhibition groups (mean ± SD).

	High inhibition	Low inhibition	*t* (34)	*p*
Interference effect	82.87 ± 30.17	285.77 ± 58.89	−13.01	< 0.001

### 3.2 Retrieval Practice Performance

A repeated‐measures ANOVA examined retrieval practice correct rates across inhibition ability and item competitive strength (Table 3). A significant effect for item competitive strength was found, *F* (2, 68) = 19.27, *p* < *0*.001, *η*_*p*_^2^ = 0.362, with high‐competitive strength items showing higher correct rates than those of medium‐ and low‐competitive strength (*p*s < 0.001). The effect for inhibition ability was nonsignificant, *F* (1, 34) = 2.26, *p* = 0.142, *η*_*p*_^2^ = 0.062, as was the interaction, *F* (2, 68) = 0.41, *p* = 0.666, *η*_*p*_^2^ = 0.012, suggesting that there is no difference in the correct rates of retrieval practice between the high‐ and low‐inhibition groups at each item competitive strength.

**TABLE 3:** The correct rates of retrieval practice of the high‐ and low‐inhibition groups (mean ± SD) (%).

	High strength	Medium strength	Low strength	*F*	*p*
High inhibition	82.41 ± 12.42	64.81 ± 13.27	65.28 ± 15.72		
Low inhibition	83.80 ± 12.61	70.83 ± 10.00	70.83 ± 13.48		
Item competitive strength				19.27	< 0.001
Inhibition ability				2.26	0.142
Item competitive strength × inhibition ability				0.41	0.666

### 3.3 Practice Effects

Table 4 shows the memory performance of the high‐ and low‐inhibition groups at different item competitive strengths.

**TABLE 4:** The recognition performance of the high‐ and low‐inhibition groups (mean ± SD) (%).

Competitive strength	Inhibition ability	Rp+	Rp−	Nrp
High	High	99.07 ± 2.69	61.11 ± 17.15	72.92 ± 16.18
	Low	96.76 ± 5.06	64.35 ± 25.85	61.11 ± 23.83
Medium	High	91.20 ± 15.52	62.96 ± 20.85	72.22 ± 16.36
	Low	92.13 ± 11.24	66.67 ± 20.21	75.69 ± 14.52
Low	High	93.52 ± 6.73	72.22 ± 18.74	77.08 ± 16.74
	Low	92.13 ± 8.80	65.74 ± 22.67	64.58 ± 23.58

At high‐competitive strength, a 2 (inhibition ability: high, low) × 2 (item type: Rp+, Nrp) repeated‐measures ANOVA indicated a significant main effect for item type, *F* (1, 34) = 80.72, *p* < 0.001, *η*_*p*_^2^ = 0.704, with Rp+ items recognized better than Nrp items, reflecting overall practice effects. The effect for inhibition ability was marginally significant, *F* (1, 34) = 4.11, *p* = 0.051, *η*_*p*_^2^ = 0.108, with the high‐inhibition group showing better memory performance. The interaction was nonsignificant, *F* (1, 34) = 1.90, *p* = 0.177, *η*_*p*_^2^ = 0.053, indicating no difference in practice effects between the high‐ and low‐inhibition groups.

At medium competitive strength, a 2 (inhibition ability: high, low) × 2 (item type: Rp+, Nrp) repeated‐measures ANOVA revealed a significant effect for item type, *F* (1, 34) = 26.21, *p* < 0.001, *η*_*p*_^2^ = 0.435, with higher recognition for Nrp items compared with Rp+ items. The effect for inhibition ability was nonsignificant, *F* (1, 34) = 0.42, *p* = 0.522, *η*_*p*_^2^ = 0.012, as was the interaction, *F* (1, 34) = 0.14, *p* = 0.715, *η*_*p*_^2^ = 0.004, indicating no differential practice effects between high‐ and low‐inhibition groups.

At low‐competitive strength, a 2 (inhibition ability: high, low) × 2 (item type: Rp+, Nrp) repeated‐measures ANOVA indicated a significant main effect for item type, *F* (1, 34) = 53.51, *p* < 0.001, *η*_*p*_^2^ = 0.611, with better recognition for Nrp items over Rp+ items. The effect for inhibition ability was nonsignificant, *F* (1, 34) = 2.74, *p* = 0.107, *η*_*p*_^2^ = 0.075, whereas the interaction effect was marginally significant, *F* (1, 34) = 3.42, *p* = 0.073, *η*_*p*_^2^ = 0.091. Simple effects analyses revealed no difference in recall for Rp+ items between high‐ and low‐inhibition groups (*p* = 0.598), but a trend toward a difference for Nrp items (*p* = 0.075).

### 3.4 Retrieval‐Induced Forgetting

**FIGURE 3:**
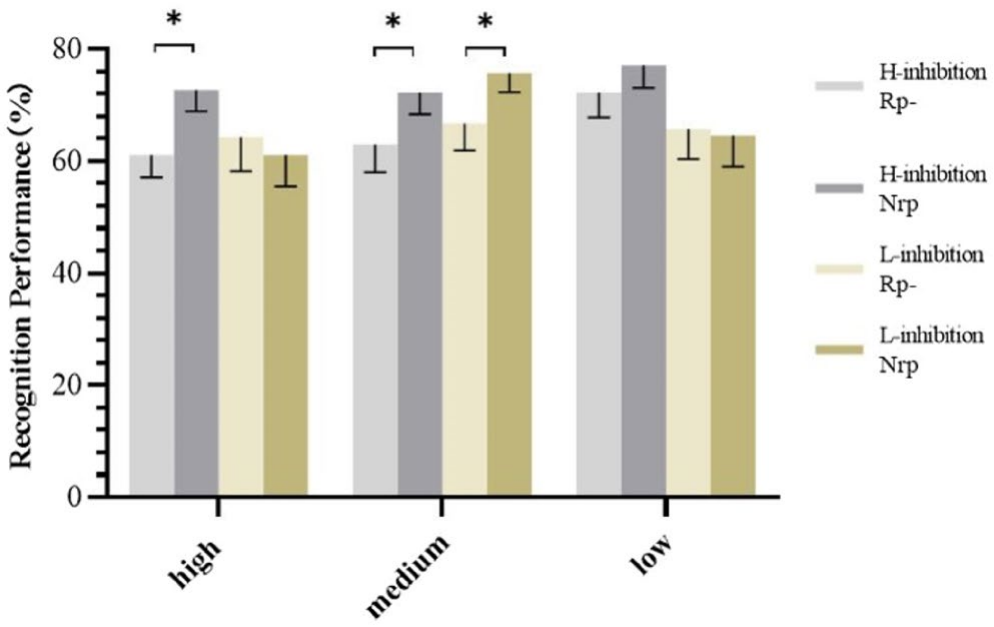
Recognition performance of Rp− and Nrp− items by item competitive strength and inhibition ability. *p < 0.05; error bars represent SE.

At high‐competitive strength, a 2 (inhibition ability: high, low) × 2 (item type: Rp−, Nrp) repeated‐measures ANOVA showed nonsignificant effects for item type (Figure 3), *F* (1, 34) = 1.17, *p* = 0.286, *η*_*p*_^2^ = 0.033 and inhibition ability, *F* (1, 34) = 0.54, *p* = 0.469, *η*_*p*_^2^ = 0.016. The interaction effect was marginally significant, *F* (1, 34) = 3.62, *p* = 0.066, *η*_*p*_^2^ = 0.096. Simple effects analyses indicated higher recognition for Nrp items than Rp− items in the high‐inhibition group, *F* (1, 34) = 4.46, *p* = 0.042, *η*_*p*_^2^ = 0.116, whereas no difference was found in the low‐inhibition group, *F* (1, 34) = 0.34, *p* = 0.566, *η*_*p*_^2^ = 0.010.

At medium competitive strength, a 2 (inhibition ability: high, low) × 2 (item type: Rp−, Nrp) repeated‐measures ANOVA indicated a significant effect for item type (Figure 3), *F* (1, 34) = 8.83, *p* = 0.005, *η*_*p*_^2^ = 0.206, with better recognition for Nrp items, indicative of RIF. The effect of inhibition ability was not significant, *F* (1, 34) = 0.47, *p* = 0.497, *η*_*p*_^2^ = 0.014. The interaction was also nonsignificant, *F* (1, 34) = 0.001, *p* = 0.970, *η*_*p*_^2^ < 0.001, suggesting no difference in the magnitude of RIF between high‐ and low‐inhibition groups.

At low‐competitive strength, a 2 (inhibition ability: high, low) × 2 (item type: Rp−, Nrp) repeated‐measures ANOVA showed no significant main effect for item type (Figure 3), *F* (1, 34) = 0.23, *p* = 0.638, *η*_*p*_^2^ = 0.007, indicating equivalent memory performance for Rp− and Nrp− items; no RIF was observed. Inhibition ability also lacked significance, *F* (1, 34) = 2.81, *p* = 0.103, *η*_*p*_^2^ = 0.076. The interaction was similarly nonsignificant, *F* (1, 34) = 0.59, *p* = 0.446, *η*_*p*_^2^ = 0.017, suggesting the absence of RIF in both high‐ and low‐inhibition groups.

Additionally, a planned comparison of RIF across high‐ and low‐inhibition groups, irrespective of item competitive strength, showed significantly higher recognition performance for Nrp items in the high‐inhibition group, *F* (1, 34) = 6.16, *p* = 0.018, *η*_*p*_^2^ = 0.153. Conversely, the low‐inhibition group exhibited no difference between Nrp and Rp− items, *F* (1, 34) = 0.20, *p* = 0.661, *η*_*p*_^2^ = 0.006. RIF was evident only in the high‐inhibition group for the overall study materials, whereas the low‐inhibition group showed no RIF.

## 4 Discussion

This study investigated the RIF in individuals with varying inhibition abilities at different item competitive strengths. Findings indicated that RIF was present only in the high‐inhibition group at high‐competitive strength, whereas the low‐inhibition group did not exhibit RIF. At medium competitive strength, both groups showed RIF without significant differences in magnitude. However, under low‐competitive strength, neither group displayed RIF. These outcomes enhance our comprehension of the interplay between inhibition ability and RIF.

First, this study's outcomes are in line with the inhibition theory of RIF, showing no RIF under low‐item competitive strength across groups with different inhibition abilities, echoing findings from Reppa et al. (2017) in object recognition. Bai and Liu (2014) and Migueles and García‐Bajos (2014) also observed RIF in high‐taxonomic frequency materials using category‐plus‐stem and cued recall, respectively. These studies collectively highlight the critical role of item competitive strength in mediating RIF's magnitude through competition dependence. In addition, studies also support competition dependence through task processes. Smith and Hunt (2000) found no RIF when participants identified differences among category items, suggesting that distinct processing reduces competition and RIF. In Shivde and Anderson (2011)'s study, a lexical decision task following word memorization revealed slower responses for semantically related words.

Despite the general support for competition dependence, some studies, such as that by Williams and Zacks (2001), have reported equal RIF effects across high‐ and low‐taxonomic frequency items. This may be due to the 8‐s study and 10‐s retrieval intervals that could have enhanced item‐cue associations, thus facilitating a blocking effect. Jakab and Raaijmakers (2009) also sought to alter competition levels based on memory performance but found no effect of competition on RIF. Their use of exemplars with moderate taxonomic frequencies and the method's validity for manipulating competition levels are subjects of debate.

Second, this study addressed a puzzle: How people with different inhibition abilities show similar RIF magnitudes in recognition tests. The broad applicability of the inhibition mechanism has led researchers to focus on the relationship between inhibition ability and RIF. According to the inhibition mechanism, when retrieving a target item, nontarget items compete for the retrieval of the target item and are thus inhibited. When the inhibition ability is stronger, nontarget items should show more impairment. Some studies have observed that individuals with different inhibition abilities exhibit equal levels of RIF. The view of *the correlated costs and benefits problem* can provide explanations for the results of those using cued recall, that is, those with low inhibition demonstrate the same level of RIF as those with high inhibition, essentially because the blocking mechanism is at work, not the inhibition mechanism at work. It remains puzzling that equivalent levels of RIF can still be observed in individuals with varying levels of inhibition when recognition tests are employed. Some researchers have argued in favor of automatic inhibition as an explanation for these findings. It suggests that the inhibition underlying RIF is an unintentional and automatic process (AhnAllen et al. 2007; Conway and Fthenaki 2003). During the retrieval of target information, individuals unintentionally and automatically inhibit irrelevant information, potentially leading to similar RIF magnitudes across different inhibition levels. However, this automatic inhibition view may not be fully convincing. First, it has been found that individuals' inhibition positively predicts the amount of the RIF effect in recognition tests (Schilling et al. 2014). Moreover, for all study materials in the present study, only the high‐inhibition group demonstrated RIF. Similarly, at high‐item competition strength, the high‐inhibition group uniquely exhibited RIF, contrasting with the lack of RIF in the low‐inhibition group. Such results, showing that the stronger the inhibition, the greater the RIF, have been supported by numerous studies (Aslan and Bäuml 2011; Román et al. 2009; Tumen and Ikier 2021). These results from both the present study and previous work obviously challenge the view of automatic inhibition.

The “competition dependence” of the inhibition mechanism has been shown to be a prerequisite for the magnitude of RIF, with the greater the competitive strength, the greater the RIF. It is likely for different inhibition individuals to demonstrate different RIF when presented with materials of different competitive strengths. The results of the present study have found RIF was influenced by the competitive strength and inhibition ability, with distinct outcomes for participants with differing inhibition abilities.

It was notable that when both high‐ and low‐inhibition groups were able to deal with competition from Rp− items at medium item competitive strength, they exhibited an equivalent magnitude of RIF. This is consistent with the results observed by previous researchers using the recognition tests (Aslan et al. 2007; Ford et al. 2004). The likely reason for this is that the competition strength generated by the nontarget items was at a moderate level at this point, and participants in both the high‐ and low‐inhibition groups were able to deal with such level of competition, and thus showed equal degrees of RIF. It may be understood that participants' inhibition ability and the item competitive strength are relative. In the retrieval practice paradigm, even low inhibitors can exhibit RIF similar to those of high inhibitors if they are able to handle competition from nontarget items.

The correct rate of retrieval practice under high‐item competitive strength was significantly higher than that under medium and low‐item competitive strength. This difference should be related to the degree of activation of items in different competition conditions. Items with higher taxonomic frequency under high‐competitive strength were more likely to be activated and successfully retrieved based on cues, compared with items with medium and low‐taxonomic frequency. Nevertheless, the accuracy of retrieval practice did not significantly differ between groups with high‐ and low‐inhibition abilities under each item competitive strength. This finding implies that the retrieval accuracy is not associated with the disparity in RIF observed between these groups.

Third, the performance of RIF should be dynamic. In the present study, the low‐inhibition group did not exhibit RIF at high‐item competitive strength. It is consistent with the findings of Liu et al. (2019), who found that at high‐competitive strength, only college students with high inhibition exhibited RIF, and college students with low inhibition showed no RIF. The RIF in the current study was expected to be generated by the inhibition mechanism using the recognition tests. The group with low inhibition demonstrated weaker cognitive inhibition and had difficulty processing interference. At high‐item competitive strength, individuals with low inhibition could not process competition from Rp− items, which resulted in them demonstrating no RIF. In short, when the competitive strength is extremely high, those with low‐inhibition ability are unable to exhibit RIF relying on the inhibition mechanism, which is in line with the inhibition mechanism. However, previous studies (Schilling et al. 2014; Storm and Bui 2016) have identified a positive correlation between inhibition ability and RIF. In these studies, individuals with higher inhibition ability tend to exhibit more robust RIF effects. This raises an important question: How can we reconcile the positive correlation between inhibition ability and RIF with the current findings, which suggest that individuals with low‐inhibition ability do not exhibit RIF in highly competitive conditions?

We propose that the relationship between inhibition ability and RIF is not a simple, linear one but rather is dynamic and context‐dependent. Cognitive inhibition is not an all‐or‐none process; rather, it operates on a continuum. In high‐item competitive strength conditions, individuals with low‐inhibition ability may struggle to inhibit competition from nontarget items, leading to a lack of RIF. However, in conditions where the competitive strength is lower or where the interference is less intense, individuals with low‐inhibition ability might still demonstrate RIF if they are able to inhibit some of the irrelevant items.

Therefore, we suggest that the inhibition mechanism underlying RIF may vary across studies and contexts. Although individuals with high‐inhibition ability consistently show RIF in high‐competition conditions, the ability of individuals with low‐inhibition ability to exhibit RIF is influenced by the specific competitive strength and the degree to which they can process and inhibit interference. As such, RIF in individuals with low‐inhibition ability may appear in studies with lower competition or with less intense interference, leading to a more dynamic interpretation of the relationship between inhibition ability and RIF.

This study has several limitations. Memory assessment was confined to correct hit rates for studied exemplars, a measure that may lack comprehensiveness. Inhibition was only measured using the Stroop task, revealing findings that correspond only to a part of the cognitive inhibition function. A variety of tasks would be needed to clarify the relationship between different inhibition processes and RIF. In addition, there was a large gender difference in the participants, predominantly female, which is one of the limitations. Future research could overcome these limitations, thereby reinforcing the study's conclusions.

## 5 Conclusion

This study provides support for competition dependence and identifies the inhibition mechanism underlying RIF. It also underscores that RIF is influenced by item competitive strength and inhibition ability, indicating individual variability in RIF across studies.

## Ethics Statement

The study was approved by the Ethical Committee of Tianjin Normal University, in accordance with the Declaration of Helsinki. Informed consent was obtained from the participants.

## Conflicts of Interest

The authors declare no conflicts of interest.

## Supporting information


Data S1.

Data S2.

Data S3.
