# Supplementary material for: Interactive Influence of Item Competitive Strength and Inhibition Ability on Retrieval‐Induced Forgetting
Source: Psych J. 2025 Mar 10;14(3):385–94. doi: 10.1002/pchj.70007 (PMC12133235; doi:10.1002/pchj.70007)
Supplement: Supplementary file 1 — Data S1. [file PCHJ-14-385-s003.pdf]

```
ONEWAY 评分 BY 竞争强度分组
  /STATISTICS DESCRIPTIVES HOMOGENEITY
  /MISSING ANALYSIS
  /POSTHOC=LSD T2 ALPHA(0.05).
```

# 单向

## 描述

评分

| | 个案数 | 平均值 | 标准差 | 标准误差 | 平均值的 95% 置信区间 | | 最小值 | 最大值 |
|---|---|---|---|---|---|---|---|---|
| | | | | | 下限 | 上限 | | |
| 1 | 24 | 6.2405 | .28399 | .05797 | 6.1205 | 6.3604 | 5.83 | 6.73 |
| 2 | 24 | 5.1302 | .51697 | .10553 | 4.9119 | 5.3485 | 3.94 | 5.85 |
| 3 | 24 | 3.6450 | .50781 | .10366 | 3.4305 | 3.8594 | 2.71 | 4.60 |
| 总计 | 72 | 5.0052 | 1.15876 | .13656 | 4.7329 | 5.2775 | 2.71 | 6.73 |

## 方差齐性检验

评分

| 莱文统计 | 自由度 1 | 自由度 2 | 显著性 |
|---|---|---|---|
| 4.709 | 2 | 69 | .012 |

## ANOVA

评分

| | 平方和 | 自由度 | 均方 | F | 显著性 |
|---|---|---|---|---|---|
| 组间 | 81.401 | 2 | 40.701 | 201.561 | .000 |
| 组内 | 13.933 | 69 | .202 | | |
| 总计 | 95.334 | 71 | | | |

# 事后检验

## 多重比较

因变量: 评分

| | (I) 竞争强度分组 | (J) 竞争强度分组 | 平均值差值 (I-J) | 标准误差 | 显著性 | 95% 置信区间 | |
|---|---|---|---|---|---|---|---|
| | | | | | | 下限 | 上限 |
| LSD | 1 | 2 | 1.11024* | .12972 | .000 | .8515 | 1.3690 |
| | | 3 | 2.59549* | .12972 | .000 | 2.3367 | 2.8543 |
| | 2 | 1 | -1.11024* | .12972 | .000 | -1.3690 | -.8515 |
| | | 3 | 1.48524* | .12972 | .000 | 1.2265 | 1.7440 |
| | 3 | 1 | -2.59549* | .12972 | .000 | -2.8543 | -2.3367 |
| | | 2 | -1.48524* | .12972 | .000 | -1.7440 | -1.2265 |
| 塔姆黑尼 | 1 | 2 | 1.11024* | .12040 | .000 | .8087 | 1.4118 |
| | | 3 | 2.59549* | .11876 | .000 | 2.2981 | 2.8928 |
| | 2 | 1 | -1.11024* | .12040 | .000 | -1.4118 | -.8087 |
| | | 3 | 1.48524* | .14792 | .000 | 1.1187 | 1.8518 |
| | 3 | 1 | -2.59549* | .11876 | .000 | -2.8928 | -2.2981 |
| | | 2 | -1.48524* | .14792 | .000 | -1.8518 | -1.1187 |

*. 平均值差值的显著性水平为 0.05。
