# Supplementary material for: Interactive Influence of Item Competitive Strength and Inhibition Ability on Retrieval‐Induced Forgetting
Source: Psych J. 2025 Mar 10;14(3):385–94. doi: 10.1002/pchj.70007 (PMC12133235; doi:10.1002/pchj.70007)
Supplement: Supplementary file 2 — Data S2. [file PCHJ-14-385-s002.pdf]

```
ONEWAY 水果 地形 犯罪 运动 疾病 职业 花卉 身体 BY group
  /STATISTICS DESCRIPTIVES HOMOGENEITY
  /MISSING ANALYSIS
  /POSTHOC=LSD T2 ALPHA(0.05).
```

单向

描述

		个案数	平均值	标准差	标准误差	平均值的 95% 置信区间 下限	上限	最小值	最大值
水果	1	12	5.8889	.38878	.11223	5.6419	6.1359	5.50	6.56
	2	12	4.7951	.45694	.13191	4.5048	5.0855	3.94	5.46
	3	12	2.7830	.91203	.26328	2.2035	3.3625	1.54	3.94
	总计	36	4.4890	1.44099	.24016	4.0014	4.9766	1.54	6.56
地形	1	12	5.7344	.41086	.11861	5.4733	5.9954	5.17	6.40
	2	12	4.4653	.33444	.09654	4.2528	4.6778	4.06	4.96
	3	12	3.3854	.44722	.12910	3.1013	3.6696	2.71	3.92
	总计	36	4.5284	1.04833	.17472	4.1737	4.8831	2.71	6.40
犯罪	1	12	6.3368	.17381	.05018	6.2264	6.4472	5.98	6.60
	2	12	5.5156	.31771	.09172	5.3138	5.7175	5.13	5.94
	3	12	4.3611	.87560	.25276	3.8048	4.9174	2.17	5.08
	总计	36	5.4045	.97861	.16310	5.0734	5.7356	2.17	6.60
运动	1	12	6.0955	.26149	.07548	5.9293	6.2616	5.83	6.73
	2	12	5.6215	.11474	.03312	5.5486	5.6944	5.44	5.79
	3	12	4.5851	.84455	.24380	4.0485	5.1217	2.90	5.35
	总计	36	5.4340	.81177	.13529	5.1594	5.7087	2.90	6.73

描述

		个案数	平均值	标准差	标准误差	平均值的 95% 置信区间 下限	平均值的 95% 置信区间 上限	最小值	最大值
疾病	1	12	6.1233	.24817	.07164	5.9656	6.2809	5.79	6.67
	2	12	5.3281	.38529	.11122	5.0833	5.5729	4.63	5.79
	3	12	3.9045	.43840	.12655	3.6260	4.1831	3.31	4.60
	总计	36	5.1186	.99644	.16607	4.7815	5.4558	3.31	6.67
职业	1	12	6.3854	.23326	.06734	6.2372	6.5336	6.13	6.73
	2	12	5.8472	.20515	.05922	5.7169	5.9776	5.48	6.08
	3	12	4.7882	.52636	.15195	4.4538	5.1226	3.88	5.40
	总计	36	5.6736	.75514	.12586	5.4181	5.9291	3.88	6.73
花卉	1	12	6.3281	.22440	.06478	6.1855	6.4707	6.04	6.75
	2	12	5.4653	.32413	.09357	5.2593	5.6712	4.85	5.85
	3	12	4.1337	.39782	.11484	3.8809	4.3864	3.54	4.75
	总计	36	5.3090	.96782	.16130	4.9816	5.6365	3.54	6.75
身体	1	12	6.2986	.10142	.02928	6.2342	6.3630	6.19	6.52
	2	12	5.6997	.20861	.06022	5.5671	5.8322	5.42	6.06
	3	12	4.8889	.28845	.08327	4.7056	5.0722	4.35	5.40
	总计	36	5.6291	.62153	.10359	5.4188	5.8393	4.35	6.52

方差齐性检验

	莱文统计	自由度 1	自由度 2	显著性
水果	11.064	2	33	.000
地形	.679	2	33	.514
犯罪	11.198	2	33	.000
运动	19.356	2	33	.000
疾病	4.036	2	33	.027
职业	8.714	2	33	.001
花卉	3.129	2	33	.057
身体	3.982	2	33	.028

ANOVA

		平方和	自由度	均方	F	显著性
水果	组间	59.567	2	29.783	74.975	.000
	组内	13.109	33	.397		
	总计	72.676	35			
地形	组间	33.177	2	16.589	103.536	.000
	组内	5.287	33	.160		
	总计	38.465	35			
犯罪	组间	23.642	2	11.821	39.499	.000
	组内	9.876	33	.299		
	总计	33.519	35			
运动	组间	14.321	2	7.160	27.027	.000
	组内	8.743	33	.265		
	总计	23.064	35			

ANOVA

		平方和	自由度	均方	F	显著性
疾病	组间	30.327	2	15.164	113.096	.000
	组内	4.425	33	.134		
	总计	34.752	35			
职业	组间	15.849	2	7.925	63.643	.000
	组内	4.109	33	.125		
	总计	19.958	35			
花卉	组间	29.333	2	14.666	140.270	.000
	组内	3.450	33	.105		
	总计	32.783	35			
身体	组间	12.014	2	6.007	131.531	.000
	组内	1.507	33	.046		
	总计	13.521	35			

事后检验

多重比较

因变量		(I) group	(J) group	平均值差值 (I-J)	标准误差	显著性	95% 置信区间 下限	95% 置信区间 上限
水果	LSD	1	2	1.09375*	.25731	.000	.5703	1.6172
			3	3.10590*	.25731	.000	2.5824	3.6294
		2	1	-1.09375*	.25731	.000	-1.6172	-.5703
			3	2.01215*	.25731	.000	1.4887	2.5356
		3	1	-3.10590*	.25731	.000	-3.6294	-2.5824
			2	-2.01215*	.25731	.000	-2.5356	-1.4887
	塔姆黑尼	1	2	1.09375*	.17319	.000	.6454	1.5421
			3	3.10590*	.28620	.000	2.3365	3.8753
		2	1	-1.09375*	.17319	.000	-1.5421	-.6454
			3	2.01215*	.29447	.000	1.2285	2.7958
		3	1	-3.10590*	.28620	.000	-3.8753	-2.3365
			2	-2.01215*	.29447	.000	-2.7958	-1.2285
地形	LSD	1	2	1.26910*	.16341	.000	.9366	1.6016
			3	2.34896*	.16341	.000	2.0165	2.6814
		2	1	-1.26910*	.16341	.000	-1.6016	-.9366
			3	1.07986*	.16341	.000	.7474	1.4123
		3	1	-2.34896*	.16341	.000	-2.6814	-2.0165
			2	-1.07986*	.16341	.000	-1.4123	-.7474
	塔姆黑尼	1	2	1.26910*	.15293	.000	.8727	1.6655
			3	2.34896*	.17531	.000	1.8958	2.8021

多重比较

因变量		(I) group	(J) group	平均值差值 (I-J)	标准误差	显著性	95% 置信区间 下限	95% 置信区间 上限
		2	1	-1.26910*	.15293	.000	-1.6655	-.8727
			3	1.07986*	.16121	.000	.6606	1.4991
		3	1	-2.34896*	.17531	.000	-2.8021	-1.8958
			2	-1.07986*	.16121	.000	-1.4991	-.6606
犯罪	LSD	1	2	.82118*	.22334	.001	.3668	1.2756
			3	1.97569*	.22334	.000	1.5213	2.4301
		2	1	-.82118*	.22334	.001	-1.2756	-.3668
			3	1.15451*	.22334	.000	.7001	1.6089
		3	1	-1.97569*	.22334	.000	-2.4301	-1.5213
			2	-1.15451*	.22334	.000	-1.6089	-.7001
	塔姆黑尼	1	2	.82118*	.10454	.000	.5445	1.0978
			3	1.97569*	.25770	.000	1.2605	2.6909
		2	1	-.82118*	.10454	.000	-1.0978	-.5445
			3	1.15451*	.26889	.002	.4250	1.8840
		3	1	-1.97569*	.25770	.000	-2.6909	-1.2605
			2	-1.15451*	.26889	.002	-1.8840	-.4250
运动	LSD	1	2	.47396*	.21013	.031	.0464	.9015
			3	1.51042*	.21013	.000	1.0829	1.9379
		2	1	-.47396*	.21013	.031	-.9015	-.0464
			3	1.03646*	.21013	.000	.6089	1.4640

多重比较

因变量		(I) group	(J) group	平均值差值 (I-J)	标准误差	显著性	95% 置信区间 下限	95% 置信区间 上限
		3	1	-1.51042*	.21013	.000	-1.9379	-1.0829
			2	-1.03646*	.21013	.000	-1.4640	-.6089
	塔姆黑尼	1	2	.47396*	.08243	.000	.2528	.6952
			3	1.51042*	.25522	.000	.8126	2.2083
		2	1	-.47396*	.08243	.000	-.6952	-.2528
			3	1.03646*	.24604	.004	.3492	1.7237
		3	1	-1.51042*	.25522	.000	-2.2083	-.8126
			2	-1.03646*	.24604	.004	-1.7237	-.3492
疾病	LSD	1	2	.79514*	.14949	.000	.4910	1.0993
			3	2.21875*	.14949	.000	1.9146	2.5229
		2	1	-.79514*	.14949	.000	-1.0993	-.4910
			3	1.42361*	.14949	.000	1.1195	1.7277
		3	1	-2.21875*	.14949	.000	-2.5229	-1.9146
			2	-1.42361*	.14949	.000	-1.7277	-1.1195
	塔姆黑尼	1	2	.79514*	.13230	.000	.4485	1.1418
			3	2.21875*	.14543	.000	1.8348	2.6027
		2	1	-.79514*	.13230	.000	-1.1418	-.4485
			3	1.42361*	.16848	.000	.9877	1.8595
		3	1	-2.21875*	.14543	.000	-2.6027	-1.8348
			2	-1.42361*	.16848	.000	-1.8595	-.9877

多重比较

因变量		(I) group	(J) group	平均值差值 (I-J)	标准误差	显著性	95% 置信区间 下限	95% 置信区间 上限
职业	LSD	1	2	.53819*	.14406	.001	.2451	.8313
			3	1.59722*	.14406	.000	1.3041	1.8903
		2	1	-.53819*	.14406	.001	-.8313	-.2451
			3	1.05903*	.14406	.000	.7659	1.3521
		3	1	-1.59722*	.14406	.000	-1.8903	-1.3041
			2	-1.05903*	.14406	.000	-1.3521	-.7659
	塔姆黑尼	1	2	.53819*	.08967	.000	.3062	.7702
			3	1.59722*	.16620	.000	1.1515	2.0429
		2	1	-.53819*	.08967	.000	-.7702	-.3062
			3	1.05903*	.16308	.000	.6183	1.4997
		3	1	-1.59722*	.16620	.000	-2.0429	-1.1515
			2	-1.05903*	.16308	.000	-1.4997	-.6183
花卉	LSD	1	2	.86285*	.13201	.000	.5943	1.1314
			3	2.19444*	.13201	.000	1.9259	2.4630
		2	1	-.86285*	.13201	.000	-1.1314	-.5943
			3	1.33160*	.13201	.000	1.0630	1.6002
		3	1	-2.19444*	.13201	.000	-2.4630	-1.9259
			2	-1.33160*	.13201	.000	-1.6002	-1.0630
	塔姆黑尼	1	2	.86285*	.11380	.000	.5658	1.1599
			3	2.19444*	.13185	.000	1.8462	2.5427

多重比较

因变量		(I) group	(J) group	平均值差值 (I-J)	标准误差	显著性	95% 置信区间 下限	95% 置信区间 上限
		2	1	-.86285*	.11380	.000	-1.1599	-.5658
			3	1.33160*	.14813	.000	.9476	1.7156
		3	1	-2.19444*	.13185	.000	-2.5427	-1.8462
			2	-1.33160*	.14813	.000	-1.7156	-.9476
身体	LSD	1	2	.59896*	.08724	.000	.4215	.7765
			3	1.40972*	.08724	.000	1.2322	1.5872
		2	1	-.59896*	.08724	.000	-.7765	-.4215
			3	.81076*	.08724	.000	.6333	.9883
		3	1	-1.40972*	.08724	.000	-1.5872	-1.2322
			2	-.81076*	.08724	.000	-.9883	-.6333
	塔姆黑尼	1	2	.59896*	.06696	.000	.4204	.7775
			3	1.40972*	.08826	.000	1.1699	1.6496
		2	1	-.59896*	.06696	.000	-.7775	-.4204
			3	.81076*	.10276	.000	.5431	1.0784
		3	1	-1.40972*	.08826	.000	-1.6496	-1.1699
			2	-.81076*	.10276	.000	-1.0784	-.5431

*. 平均值差值的显著性水平为 0.05。
